# Sex Differences in Behavioral Sensitivities After Traumatic Brain Injury

**DOI:** 10.3389/fneur.2020.553190

**Published:** 2020-11-25

**Authors:** Ann N. Hoffman, Sonya L. Watson, Anna S. Makridis, Anisha Y. Patel, Sarah T. Gonzalez, Lindsay Ferguson, Christopher C. Giza, Michael S. Fanselow

**Affiliations:** ^1^Neurosurgery, Brain Injury Research Center, University of California, Los Angeles, Los Angeles, CA, United States; ^2^Psychology, University of California, Los Angeles, Los Angeles, CA, United States; ^3^University of California, Los Angeles Steve Tisch BrainSPORT Program, Los Angeles, CA, United States; ^4^Staglin Center for Brain and Behavioral Health, University of California, Los Angeles, Los Angeles, CA, United States; ^5^Division of Neurology, Department of Pediatrics, University of California, Los Angeles Mattel Children's Hospital, Los Angeles, CA, United States; ^6^Department of Psychiatry and Biobehavioral Science, Los Angeles, CA, United States

**Keywords:** migraine, PTSD–post-traumatic stress disorder, traumatic brain injury, sensory sensitivity, defensive behavior, fear & anxiety, sex differences

## Abstract

Traumatic brain injury (TBI) is associated with high rates of post-injury psychiatric and neurological comorbidities. TBI is more common in males than females despite females reporting more symptoms and longer recovery following TBI and concussion. Both pain and mental health conditions like anxiety and post-traumatic stress disorder (PTSD) are more common in women in the general population, however the dimorphic comorbidity in the TBI population is not well-understood. TBI may predispose the development of maladaptive anxiety or PTSD following a traumatic stressor, and the impact of sex on this interaction has not been investigated. We have shown that white noise is noxious to male rats following fluid percussion injury (FPI) and increases fear learning when used in auditory fear conditioning, but it is unclear whether females exhibit a similar phenotype. Adult female and male rats received either lateral FPI or sham surgery and 48 h later received behavioral training. We first investigated sex differences in response to 75 dB white noise followed by white noise-signaled fear conditioning. FPI groups exhibited defensive behavior to the white noise, which was significantly more robust in females, suggesting FPI increased auditory sensitivity. In another experiment, we asked how FPI affects contextual fear learning in females and males following unsignaled footshocks of either strong (0.9 mA) or weaker (0.5 mA) intensity. We saw that FPI led to rapid acquisition of contextual fear compared to sham. A consistent pattern of increased contextual fear after TBI was apparent in both sexes across experiments under differing conditioning protocols. Using a light gradient open field task we found that FPI females showed a defensive photophobia response to light, a novel finding supporting TBI enhanced sensory sensitivity across modalities in females. General behavioral differences among our measures were observed between sexes and discussed with respect to interpretations of TBI effects for each sex. Together our data support enhanced fear following a traumatic stressor after TBI in both sexes, where females show greater sensitivity to sensory stimuli across multiple modalities. These data demonstrate sex differences in emergent defensive phenotypes following TBI that may contribute to comorbid PTSD, anxiety, and other neurological comorbidities.

## Introduction

Traumatic brain injuries (TBI) affect an estimated 2.8 million people in the United States every year (1). Following TBI highly prevalent comorbid conditions emerge that affect mental health including anxiety and stress related disorders like post-traumatic stress disorder (PTSD). This is especially the case with less severe brain injuries. Thus, TBI has far reaching negative effects on overall health and quality of life (2, 3). It is well-known that the overall prevalence of TBI is higher in males than females (1, 4–7). However, when risk exposures are controlled for, such as in sports and athletics, females sustain TBI, and concussion more often than males (8, 9). Women are also more likely than men to sustain injuries from assault or interpersonal violence (10, 11), an understudied population that often endure comorbidities associated with stress and trauma (12). Multiple studies report that females have increased symptom severity as well as longer recovery profiles than males after sports concussion (13–16). Historically, the majority of clinical trials (17) and neuroscience, and biomedical research (18, 19) on TBI pathophysiology and functional outcomes have focused primarily on male subjects (20). Despite a paucity of research on females, emerging research is beginning to reveal sex differences in fundamental mechanisms of injury and consequences of TBI (21), including differences in axon structure following stretch injury (22), as well as post-TBI neuroinflammation (23). With the prevalence of TBI and comorbid complications on the rise, we have a large gap to fill in our understanding of the impact of TBI and sex on behavioral and neurological comorbidities and respective pathophysiology.

In the general population, some psychiatric, and mood disorders that affect emotion and defensive behavior are more prevalent in women than in men (24–26). In particular, anxiety disorders are 1.5–2 times (26), and stress and trauma related disorders like PTSD are at least 2 times more common in women than in men (27). Other neurologic conditions such as pain disorders and migraine are more prevalent in women (28). The aforementioned health and mental health conditions are often affected by and comorbid with TBI, however less is known about the sexually dimorphic comorbidities following TBI. For humans, although male gender is a known risk factor for TBI (4), female gender may be considered at increased risk for complicated comorbidities that affect sensory, pain, and psychological health. Animal models of TBI offer a controlled, prospective approach to study effects of TBI using sex as a biological variable to address these questions by investigating changes in conserved defensive behaviors related to fear and anxiety.

TBI may predispose the development of maladaptive anxiety or PTSD following a subsequent traumatic stressor, however the impact of sex on this interaction has not been previously studied. Defensive behaviors such as freezing in response to aversive and fearful stimuli are hardwired and conserved across species, including humans (29). Pre-clinical studies identifying conditions that heighten defensive behaviors in animals help establish models that allow us to investigate the underlying mechanisms that may be present in human psychiatric conditions associated with maladaptive fear and anxiety. We have shown enhanced fear in male rats following lateral fluid percussion injury (FPI) (30, 31), and that auditory sensitivity may underlie the vulnerability of TBI on enhanced fear (30). It is unclear whether females exhibit a similar sensitivity and enhanced fear phenotype, and also whether the stimulus sensitivity after TBI occurs across other modalities such as with light in photophobia. In the current study we asked whether females respond to stressful stimuli differently after TBI and how this may impact fear learning and anxiety-like behavior. Such differences could contribute to sex differences in psychiatric comorbidities after TBI, which could influence their clinical presentation and management.

## Materials and Methods

### Subjects

Young adult female and male Sprague-Dawley rats (Envigo; 9–10 weeks upon arrival) were pair housed with same sex cage mates and maintained on a 12 h light/dark cycle with food and water *ad libitum*. All experiments were performed during the light phase of the light cycle. Prior to surgery, all rats were handled approximately 1 min/day for 4 days. Naturally cycling females were used in all experiments and estrus phase was not monitored to avoid any confounding influence of additional handling after the start of the experiment. Within each sex, animals were randomized for injury condition and conditions were counterbalanced across testing chambers when applicable. Body weights ranged from ~180–250 g for females and ~280–400 g for males across experiments. All procedures were conducted with approval from the University of California Los Angeles Institutional Care and Use Committee and Use of Laboratory Animals (protocol #2008-038).

### Lateral Fluid Percussion Injury

**Figure 1:**
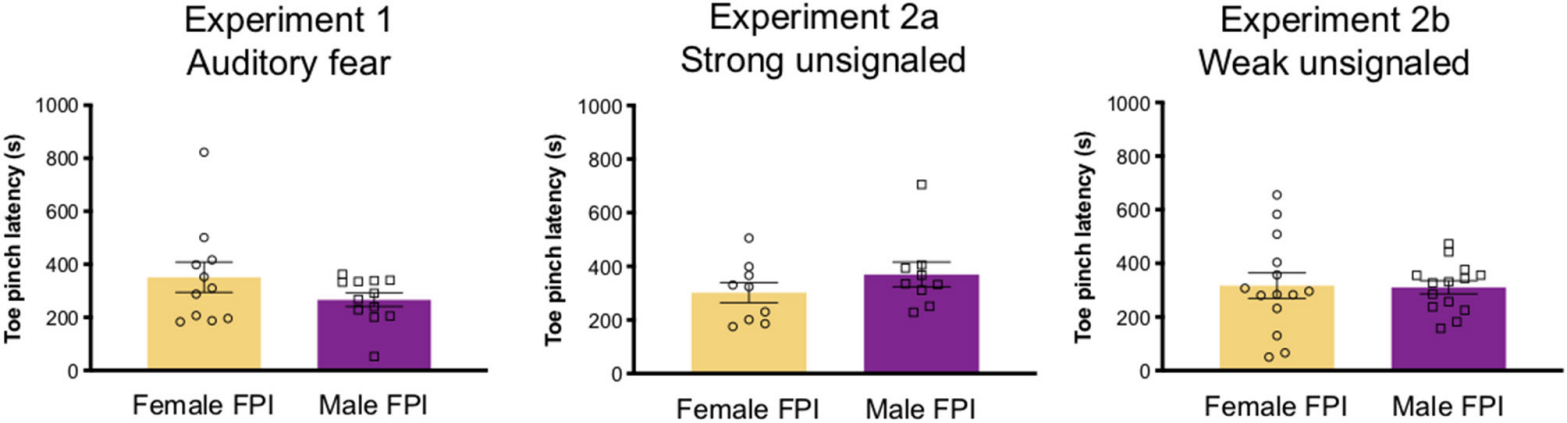
Injury severity across experiments. Although drop angle was adjusted between sexes in lateral fluid percussion injury settings (angle 13 for females, 14 for males), injury severity was balanced across sexes for all experiments as measured by latency for toe pinch withdrawal after impact. Data are represented as mean ± SEM; *n* = 9–14/group depending on the experiment.

Rats underwent either sham surgery or mild-moderate lateral fluid percussion injury (FPI). Lateral FPI is a general brain movement injury that exposes the entire brain to forces generated by the percussion (32–34). FPI was induced using a previously published protocol (31, 35–37) typically used in our laboratory. Animals were anesthetized under a 1–2% isoflurane-oxygen mixture and secured in a stereotaxic frame. A midline incision was made followed by a left hemisphere 3 mm diameter craniotomy centered 3 mm posterior and 6 mm lateral to bregma. A plastic injury cap was adhered to the skull with silicone gel and dental cement. When dental cement was dry and the injury cap secure, the cap was filled with sterile saline and the animal was removed from anesthesia. The injury cap was attached to the fluid percussion injury device (Virginia Commonwealth University, Richmond, Virginia). Upon toe pinch response, a brief fluid pulse (~20 ms) of saline was administered directly to the dura. The impact is caused by a pendulum drop from a controlled height to impact the piston of a fluid filled reservoir, forcing the brief fluid pulse in the cranial cavity through the cap (32). Apnea and return of reflex as measured by latency to limb withdrawal following toe pinch were measured to determine injury severity. In order to balance injury severity between females and males in the FPI groups, we adjusted the drop angle on the fluid percussion injury device to 13 for females, where we use 14 in males to produce a comparable injury severity by average toe pinch latency (see Figure 1). Injury severity in the mild-moderate range was used in this study. There was no difference between sexes across experiments for injury severity as a measure of toe pinch withdrawal [Experiment 1: t(22) = 1.43, *p* = 0.166; Experiment 2: t(18) = 0.7, *p* = 0.49; Experiment 3: t(26) = 1.13, *p* = 0.094; see Figure 1]. Furthermore, there was no difference in the atmospheres of pressure (atm) produced by the injury device when the drop angle was modified for females; Experiment 1: females, 2.66 ± 0.28; males, 2.66 ± 0.21; t(22) = 0.034, *p* = 0.97; Experiment 2: females 2.65 ± 0.28; males, 2.74 ± 0.14; t(18) = 0.858, *p* = 0.4; Experiment 3: females, 2.44 ± 0.41; males 2.62 ± 0.46; t(25) = 1.062, *p* = 0.3. Immediately following the toe pinch response, rats were then placed back on anesthesia to remove the injury cap and suture the scalp. Sham animals received the same surgical procedures except for the fluid pulse impact. Upon completion of surgery, animals were placed in a heated recovery chamber until normal behavior resumed and returned to the vivarium. Animals were weighed and monitored post-operatively for a week after surgery or until the end of the experiment.

### Experiment 1: Phonophobia and Auditory Fear Conditioning

Our previous study revealed that 48 h following lateral FPI, adult male rats exhibited increased defensive behavior (freezing) when exposed to 75 dB white noise prior to fear conditioning with mild shocks (30); however, it is unknown if females display a similar phonophobia-like phenotype after FPI. Experiment 1 consisted of a series of behavioral tests related to auditory sensitivity and signaled fear conditioning. We tested for FPI and sex effects on phonophobia, auditory fear conditioning, recent and remote context fear as well as auditory fear memory to trained and novel auditory stimuli. We tested remote fear memory 4 weeks after FPI and fear conditioning to determine the lasting effects of TBI on fear. As in our previous study, behavioral testing began 48 h following FPI or sham surgeries. Four identical fear conditioning chambers equipped with the Med Associates Video Freeze system were used for behavioral training and testing (30 × 25 × 25 cm, MedAssociates; Fairfax, VT). Two distinct contexts were used for fear conditioning and testing (Context A and Context B) that differed in transport mode (uncovered home cage or opaque plastic tub), physical room location, room and test chamber lighting condition (on or off), tactile cues (shock grid vs. smooth floor), and test chamber scent (50% windex or 1% acetic acid). Percent time freezing, used as a measurement of fear, was scored automatically by VideoFreeze software set to a threshold that was calibrated to a highly trained observer (MSF). In rats and other species, freezing is the dominant defensive response upon detection of a predator, and is activated by learned fear (38). For shock reactivity, average motion index was scored during each 2 s shock period calculated as a measure of pixel change from background. Rats were transported and placed in a novel context chamber (Context A). Following a 3-min baseline period, all animals were exposed to seven 30 s presentations of 75 dB white noise with 120 s inter trial intervals (ITIs). Percent time freezing during and in between noise trials was measured. The next day, all rats were placed back in Context A and fear conditioned to the same auditory cue. Fear conditioning consisted of 10 trials of 30 s/75 dB white noise followed by a 2 s/0.9 mA footshock. Trials were presented at a fixed interval with 120 s ITIs. One day after the white noise-shock signaled fear conditioning, all subjects were again transported and placed back into Context A for 8 min with no auditory stimuli and tested for recent context fear. Following these procedures rats were returned to the vivarium and were left undisturbed for 4 weeks aside from standard husbandry procedures. At the end of the 4 week period all animals were handled briefly for 4 days and re-acclimated to transport procedures on the last 2 days (transported to Context A room and left for 10 min before return to vivarium). All rats were then tested for remote context fear by being placed back into Context A chamber for 8 min. To test for auditory fear memory, the next day all subjects were placed into a novel context (Context B) for a 15 min pre-exposure to reduce the influence of context generalization. The following day subjects were re-exposed to Context B and after a 3 min baseline period, were tested for cue fear memory with 4 trials of the trained auditory cue 30 s/75 dB white noise. We also tested auditory fear generalization to a novel cue. Rats were given a single 15 min context extinction session in Context B to decrease the influence of fear from the trained cue test from the previous day. The following day, in Context B rats were tested for tone fear generalization with 4 trials of an untrained, novel tone of the same intensity (30 s/2,800 Hz/75 dB pure tone). Experimental procedures for Experiment 1 are outlined in Table 1.

**Table 1:** Experiment 1 timeline and description.

**Day (from FPI)**	**Task (Context)**	**Rationale**
−4 to −1	Handling	
0	Mild-moderate FPI	TBI
2	White noise pre-exposure (A)	Phonophobia test
3	White noise-shock fear conditioning (A)	Traumatic event
4	Context test (A)	Recent context fear memory
5–30	Rest	
31	Context test (A)	Remote context fear memory
32	Pre-exposure context (B)	
33	White noise test (B)	Trained cue fear memory
34	Context extinction (B)	
35	Tone test (B)	Generalized fear

### Experiment 2: Context Fear

It is well-documented that female rats and mice tend to display less contextual fear when compared to males (39–42). There is also evidence that female and male rats may have different shock sensitivities (43). Previous work from our lab has reported that FPI enhanced fear learning to context when footshocks were signaled by white noise (30, 31), but had no effect when shocks are unsignaled during training (31). To get a better picture of how shock intensity and sex impact fear after TBI, in experiment 2 we investigated how FPI affects contextual fear following both strong (Experiment 2A; 0.9 mA) and weak (Experiment 2B; 0.5 mA) unsignaled shocks. Forty young adult female and male Sprague Dawley rats (9–10 weeks old upon arrival) were acclimated to the vivarium and briefly handled daily for 4 days prior to mild-moderate FPI or sham surgery. Two days after surgery, all subjects were fear conditioned with unsignaled foot shocks in a novel context chamber. Animals were placed in the chamber and following an initial baseline period of 210 s were presented with 10 trials of 2 s/0.9 mA unsignaled footshocks. Shocks were delivered at a fixed interval with 2 min between trials. Context fear acquisition was measured as percent time freezing during the 30 s interval prior to each shock onset. The next day, all animals were placed back in the context for 15 min and tested for contextual fear memory.

To further test differences in shock sensitivity and tease out the potential ceiling effects in context fear in males, for experiment 2B an additional cohort was run using the same handling, surgery, and conditioning protocols to test acquisition of context fear in response to weaker shocks of 0.5 mA, and tested in an 8 min context test.

### Experiment 2: Anxiety-Like Behavior and Photophobia

A subset of the animals from the weak shock cohort (Experiment 2B; *n* = 9–10/group) were tested on an additional task for anxiety-like behavior and photophobia in a modified open field task with light gradient. The light gradient open field task was used to measure classical anxiety-like behaviors (locomotion, velocity, thigmotaxis) (44–46) with the addition of the sudden onset of bright light at one end of the arena that causes an activity response to the change in environmental conditions (47, 48). This task also offers a novel way to measure photophobia, or sensitivity to light, by measuring the amount of time the animal spends in the zone farthest from the light source. The rectangular open arena (46 × 86 × 30 cm) was situated in a dark room lit with red lights. Three lamps were positioned outside each end of the arena (6 total), facing down as to not directly illuminate the inside of the arena. LED bulbs were used to maintain temperature during the light condition on the lit side of the arena. An overhead camera sensitive to an infared light in the room recorded animal behavior throughout the task onto a computer outside the testing room and video was analyzed offline via Ethovision software (Noldus; Leesburg, VA). The rectangular arena was divided into 4 equivalent zones, where during the light on phase of the task, zone 1 was the brightest and closest to the lamps, zone 4 was the darkest on the distal end of the lamps and zones 2 and 3 were of descending illumination along the gradient (see Figure 6G). A light meter placed in the center of each zone measured illumination in the light on condition where zone 1 was 2,160 lux, zone 2 was 840 lux, zone 3 was 420 lux and zone 4 was 260 lux. In the dark, the open area was 0 lux. Average velocity and time spent in zones were analyzed across the 12 min task. In this task, rats were placed in the center of the arena and allowed to explore the area in the dark for 4 min. After 4 min, the arena was illuminated by the lamps situated outside one side of the arena creating a light gradient across the arena. Rats explored the arena during the light on phase for 4 min before the light was then turned off and left for an additional 4 min before the animal was removed at the end of the 12 min task. The lighted side during the light-on phase was counterbalanced across trials and conditions to eliminate any bias of side preference. An experimental timeline for Experiment 2 is outlined in Table 2.

**Table 2:** Experiment 2 timeline and description.

**Day (from FPI)**	**Task**	**Rationale**
−4 to −1	Handling	
0	Mild-moderate FPI	TBI
2	Unsignaled strong (expt 2A, 0.9 mA) or weak (expt 2B, 0.5 mA) shocks fear conditioning (A)	Traumatic event (different intensities)
3	Context test (A)	Context fear memory
4*	Light gradient open field (*subset cohort from expt 2B weak shocks, *n* = 9–10/group)	Photophobia

### Data Analysis

Behavioral data were analyzed using either two way or mixed factors analysis of variance (ANOVA) for sex (female, male), injury group (sham, FPI), and time or trials where appropriate. Specific analyses are described in each results section. Statistical significance was determined at a *p*-value of 0.05 or less, and when significant interactions were detected, *post hoc* contrasts were performed for simple main effects.

## Results

### Experiment 1

**Figure 2:**
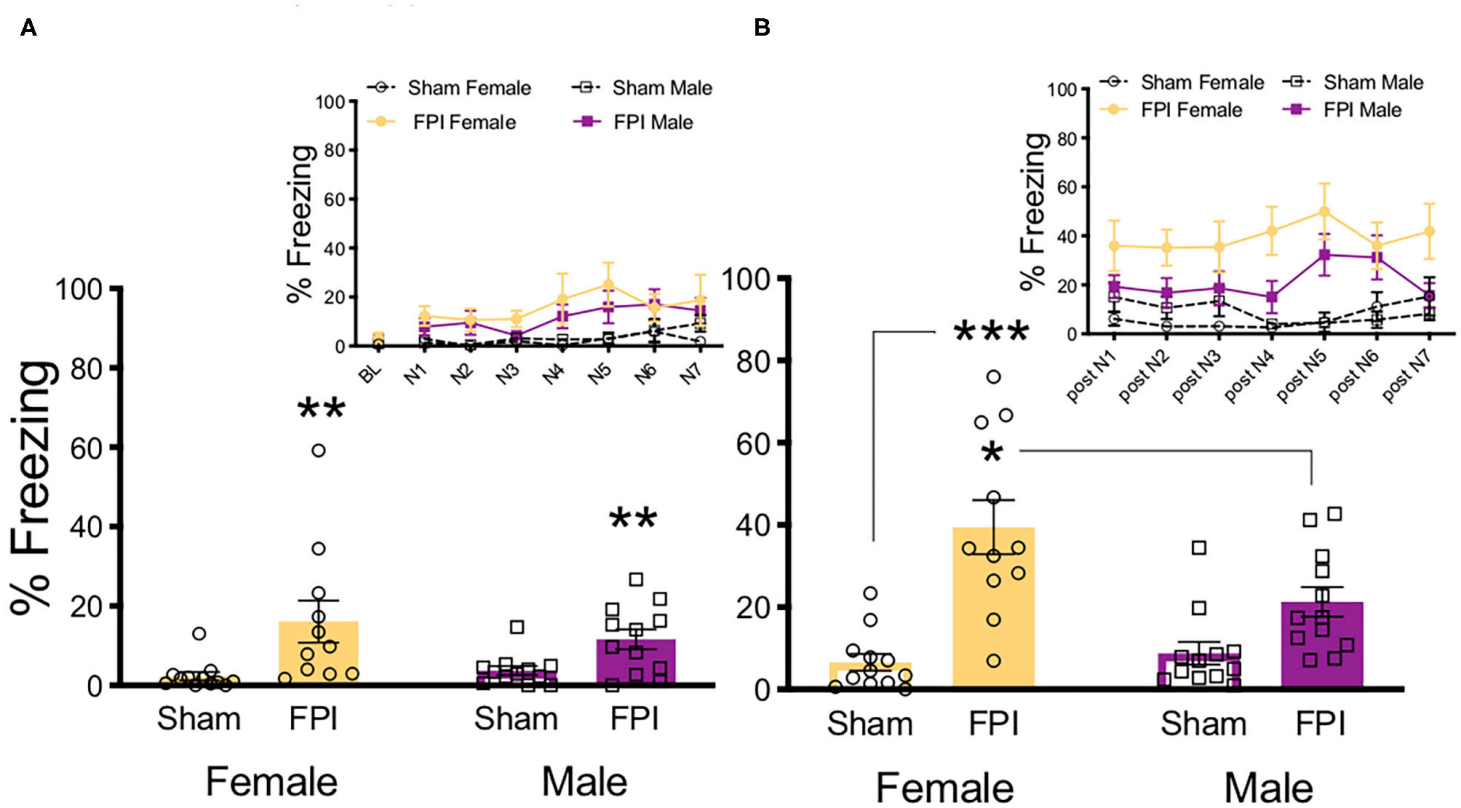
Females exhibit more phonophobia following FPI. **(A)** Average freezing across trials of 30s/75 dB white noise pre-exposure. Inset depicts freezing across 7 white noise trials. FPI increased freezing during white noise trials, regardless of sex; **p = 0.001. **(B)** Average freezing during 30 s post noise interval, inset depicts freezing during inter trial intervals. FPI females displayed the highest magnitude off fear following noise termination, compared to Female Sham ***p < 0.001, and even FPI Male *p = 0.021. Data are represented as mean ± SEM; *n* = 11–12/group.

To determine sex differences in auditory sensitivity due to FPI, injured and sham animals of both sexes were exposed to white noise alone (7 trials/75 dB/30 s) and freezing was measured. Levels of freezing were evaluated across groups during white noise exposure and during ITIs following noise offset (One FPI female was lost to mortality from the impact; group sizes from three replicated surgery cohorts include: Sham Female, *n* = 12; FPI Female, *n* = 11; Sham Male, *n* = 12; FPI Male, *n* = 12). As shown in Figure 2A, during white noise trials both female and male FPI groups had increased levels of freezing compared to their respective sham groups, resulting in an overall effect of injury [main effect of FPI: F(1,43) = 14.079, *p* = 0.001] but not sex [F(1,43) = 0.282, *p* = 0.589] as determined by a mixed factors ANOVA for sex, injury, and across trials. When observing freezing during ITIs, as seen in Figure 2B, there was a sex × injury interaction [F(1,43) = 6.596, *p* = 0.014]. Interestingly, the female FPI group displayed the highest magnitude of freezing between white noise trials compared to all other groups (FPI female vs. Sham female [F(1,21) = 24.631, *p* < 0.001]; FPI female vs. FPI male [F(1,21) = 6.215, *p* = 0.021).

**Figure 3:**
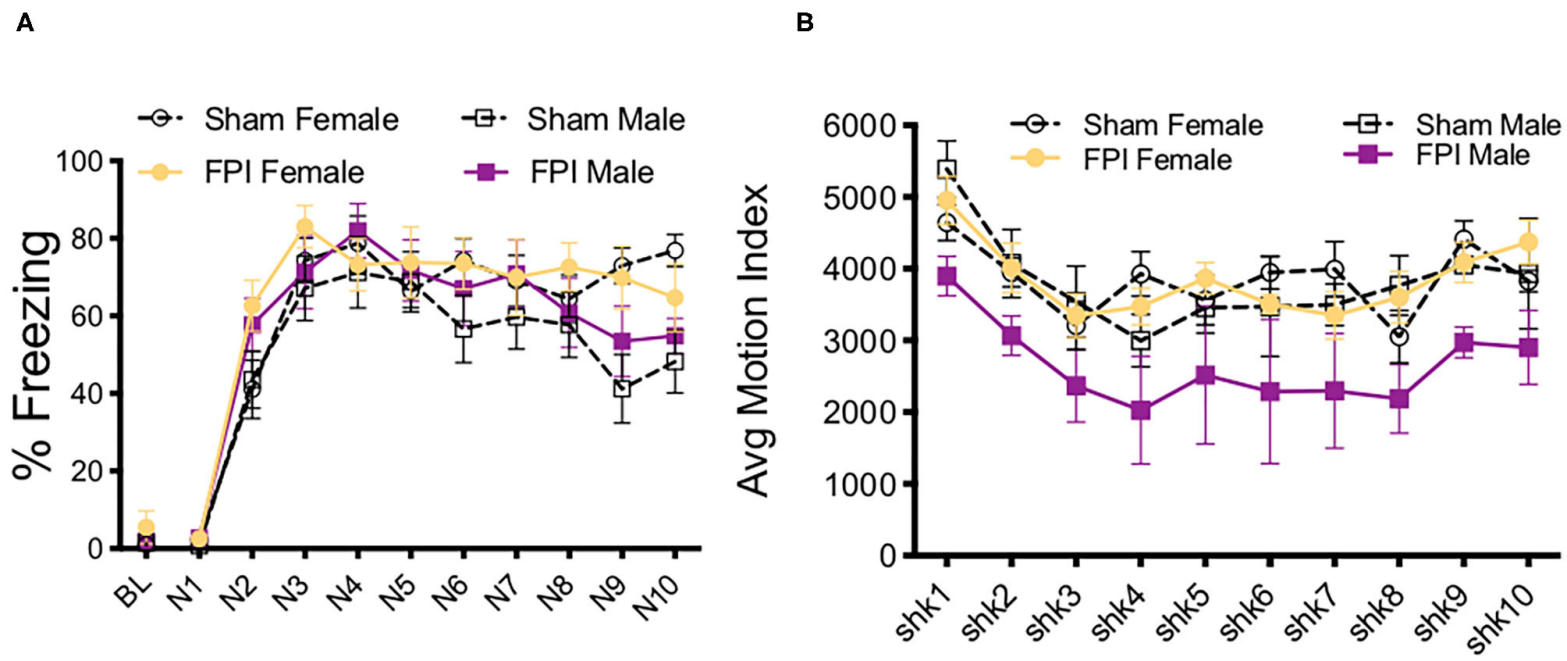
White noise-shock fear conditioning as a traumatic stressor following FPI. **(A)** No differences in baseline freezing prior to white noise-shock fear conditioning. All groups increased freezing across conditioning trials, with no differences between sex or injury conditions. **(B)** Reactivity to shock across fear conditioning trials. Although FPI male rats tended to exhibit reduced shock reactivity across noise-shock conditioning trails, the average motion index during shock did not differ significantly across sex or injury group. Data are represented as mean ± SEM; *n* = 11–12/group.

Following white noise pre-exposure, levels of auditory fear conditioning acquisition were examined across the four groups; baseline freezing, shock reactivity, and freezing levels across trials of white noise-shock pairings (10 trials/75 dB/30 s followed by 0.9 mA/2 s shock). Baseline freezing levels in the pre-exposed context were evaluated, no significant differences or interactions across any groups were found (Figure 3A). During white noise-shock pairings there was a strong trend toward an effect of sex where males tended to freeze less across conditioning trials [F(1,43) = 3.933, *p* = 0.054) and there was no significant effect of injury nor an interaction between factors (Figure 3A). Although FPI male rats tended to exhibit reduced shock reactivity across noise-shock conditioning trials, the average motion index during shock did not differ significantly across sex or injury group (Figure 3B).

**Figure 4:**
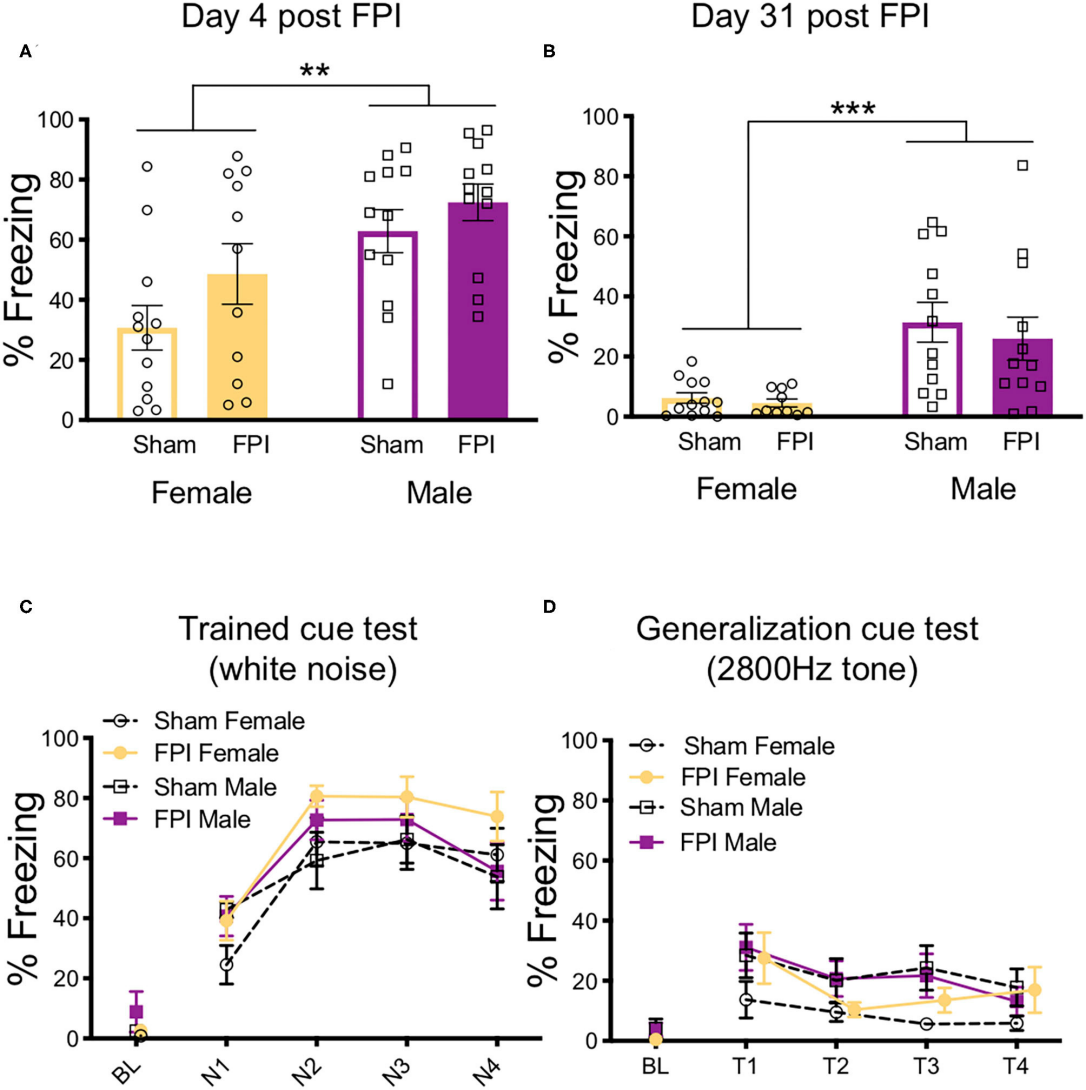
Females have reduced contextual fear at recent and remote time points after auditory fear conditioning. **(A)** 24 h after white noise-shock fear conditioning, (4 days post FPI), females displayed less fear to the context than males, regardless of FPI; **p = 0.001. **(B)** Four weeks later, when tested for remote context fear (31 days post FPI), females showed little if any freezing to context and therefore less compared to males, regardless of FPI; ***p < 0.001. **(C)** 33 days post injury in a novel context, all groups showed intact fear to the trained white noise cue, with no effects of injury or sex. **(D)** 35 days post injury when tested for generalized auditory fear, there were no observed effect of sex or injury. Data are represented as mean ± SEM; *n* = 10–12/group.

Context fear memory was evaluated across groups at both recent (the next day) and remote (4 weeks later) timepoints. One day post-training, males had an overall higher percentage of freezing to context than females [F(1,43) = 13.195, *p* = 0.001), although there was no main effect of injury or interaction (Figure 4A). One FPI female was lost to continued weight loss during remote recovery period, FPI female group was reduced to n=10 for remote behavior testing (Figures 4B–D). Animals were placed back into the same training context (Context A) 4 weeks later and tested for remote context fear. During the remote context fear test, we again observed a main effect of sex [F(1,43) = 20.653, *p* < 0.001) where males showed higher levels of freezing compared to females, which were considerably lower if not eliminated (Figure 4B). At the remote timepoint there was no effect of injury and no interaction.

After pre-exposure to a new context (Context B), fear memory for the trained cue (4 trials/75 dB/30 s white noise) was obtained across injury group and sex. Importantly, there was no generalized fear to Context B as indicated by no differences and near zero levels of freezing during baseline prior to cue onset. Across the four test trials there was a trial x sex interaction [F(3,126) = 2.981, *p* = 0.034]. Upon further inspection, females froze slightly more on trial 2 compared to males (Figure 4C). No significant differences were detected between FPI and sham groups during the trained cue white noise test. These data from the trained cue test suggest that all groups had intact fear memories from the fear conditioning 4 weeks prior.

To assess auditory fear generalization, after an additional exposure to Context B to reduce the influence from the previous test day, animals were again placed back into Context B and exposed to a novel untrained tone of the same intensity (4 trials/75 dB/2,800 Hz/30 s). Once again, no significant differences were found in baseline freezing levels. When analyzing the differences in freezing levels across groups, there was a significant effect of trial [F(3,126) = 6.175, *p* = 0.001], indicating that freezing decreased across generalization trials for all groups (Figure 4D). There was a near significant increase in tone freezing in females compared to males, however this did not reach statistical significance [main effect of sex: F(1,43) = 3.755, *p* = 0.059]. No other effects were significant.

### Experiment Two

#### Experiment 2A Strong Shocks

**Figure 5:**
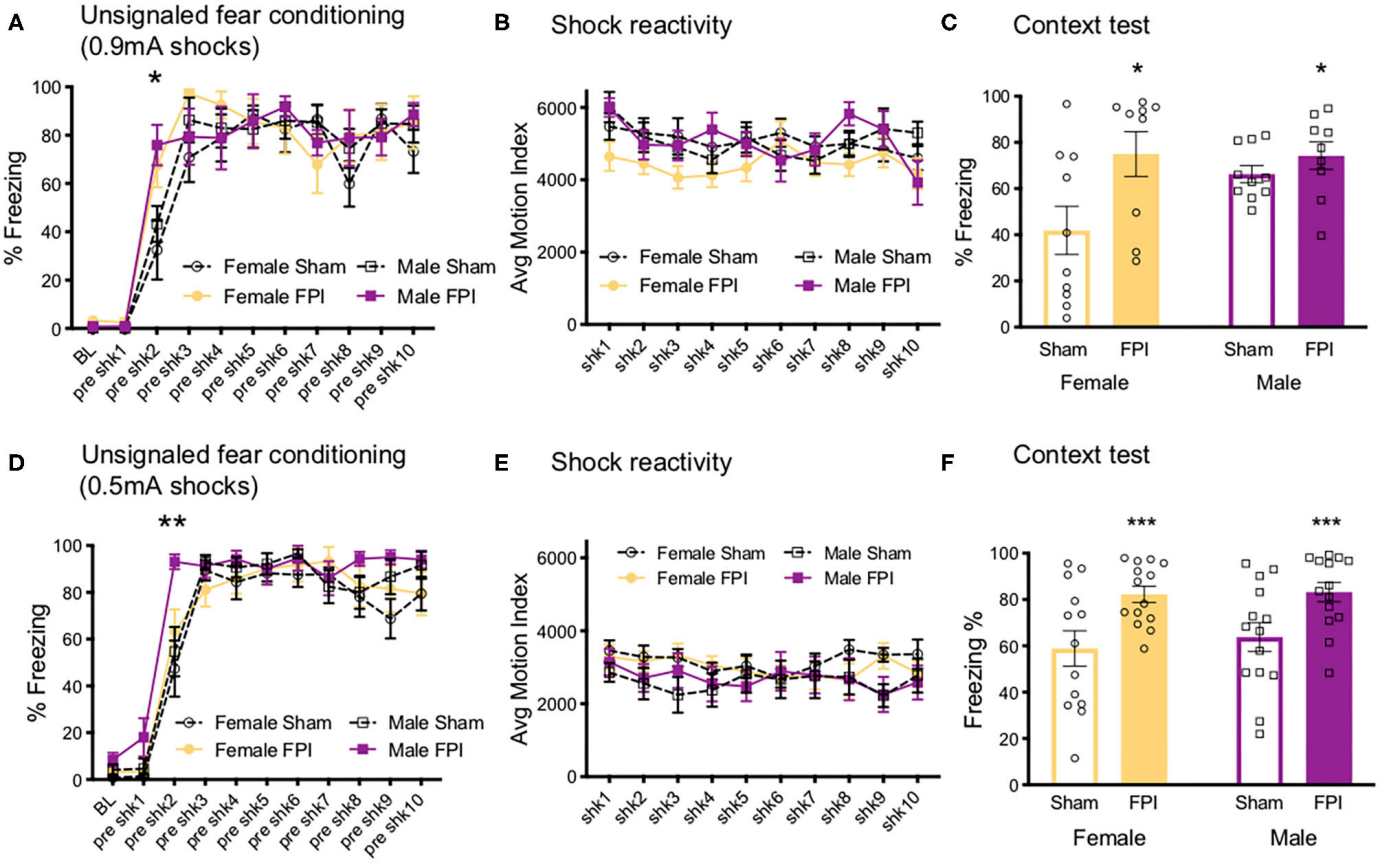
Sex and FPI effects on strong and weak unsignaled (context) fear conditioning. **(A)** Fear acquisition across 10 strong (0.9 mA) unsignaled footshocks. An injury × trial interaction revealed that FPI groups started freezing immediately following the first shock compared to sham (*p < 0.05 Sham vs. FPI). **(B)** No differences in shock reactivity during conditioning to 0.9mA shocks. **(C)** FPI groups displayed a significant increase in context fear when tested the next day (15 min test; *p < 0.05 Sham vs. FPI). **(D)** Similar to 0.9 mA shocks, FPI groups displayed an increase in freezing following the first footshock, even at a lower intensity at 0.5 mA (**p < 0.01 vs. Sham). **(E)** No differences in shock reactivity during conditioning to 0.5 mA shocks. **(F)** FPI groups showed elevated context fear compared to sham following weak unsignaled shock fear conditioning (***p < 0.01 Sham vs. FPI). Data are represented as mean ± SEM; *n* = 9–14/group.

We next investigated how contextual fear conditioning to unsignaled footshocks may be affected differently by TBI in both sexes. One FPI female and one FPI male were lost to mortality following impact, group sizes from two replicated surgery cohorts include: Sham Female, *n* = 10; FPI Female, *n* = 9; Sham Male, *n* = 10; FPI Male, *n* = 9. Both female and male, sham and FPI groups received context fear conditioning to strong, 0.9 mA unsignaled footshocks in a novel environment. We measured freezing during the 30 s interval prior to each footshock to determine whether sex and FPI had an impact on context fear acquisition. A mixed factors ANOVA revealed a trial x injury interaction where FPI groups displayed increased freezing early in the session following the first footshock (preshock interval 2; sham vs. FPI [t(36) = 3.602, *p* = 0.001; Figure 5A]. There was a main effect of trial for shock reactivity where all groups, regardless of sex or injury, showed a slight but statistically significant reduction in average motion across the 10 strong shock trials [F(9, 306) = 3.182, *p* = 0.001, Figure 5B]. A two way ANOVA for sex and FPI across the average freezing revealed a significant effect of FPI, where both female and male FPI groups showed increased freezing in the conditioning context, [F(1,34) = 6.649, *p* = 0.014; Figure 5C].

#### Experiment 2B Weak Shocks

We first aimed to determine whether and how sex impacts contextual fear conditioning following FPI. Under the strong shock protocol with 10 trials of 0.9 mA, we found that both FPI groups showed increased freezing to the context, with both groups of male rats freezing near ceiling (sham male 66.3 ± 11.5%, FPI male 74.6 ± 17.9%). Therefore, in a separate experiment, we used a weaker shock (0.5 mA) to eliminate any ceiling effects. Three replicated surgery cohorts were used in fear conditioning experiments and analysis, one female was lost to surgical complications; group sizes for the fear conditioning data were Sham Female, *n* = 13; FPI Female, *n* = 14; Sham Male, *n* = 14; FPI Male, *n* = 14. Using the same unsignaled shock protocol as the previous experiment, female and male, sham or FPI rats received 10 unsignaled 0.5 mA footshocks in a novel context. Similar to the strong shock experiment, we found a significant trial x injury interaction [F(9,459) = 2.252, *p* = 0.018], where FPI groups showed increased freezing after the first footshock [pre-shock interval 2; t(53) = 2.81, *p* = 0.007; Figure 5D]. There were no differences for sex or injury on shock reactivity Figure 5E. The next day all groups were tested for context fear. A two way ANOVA for the mean of the 8 min test revealed a significant main effect of injury [F(1,51) = 14.95, *p* < 0.001], where both female and male FPI groups showed increased freezing to the context relative to sham (Figure 5F).

#### Light Gradient Open Field

**Figure 6:**
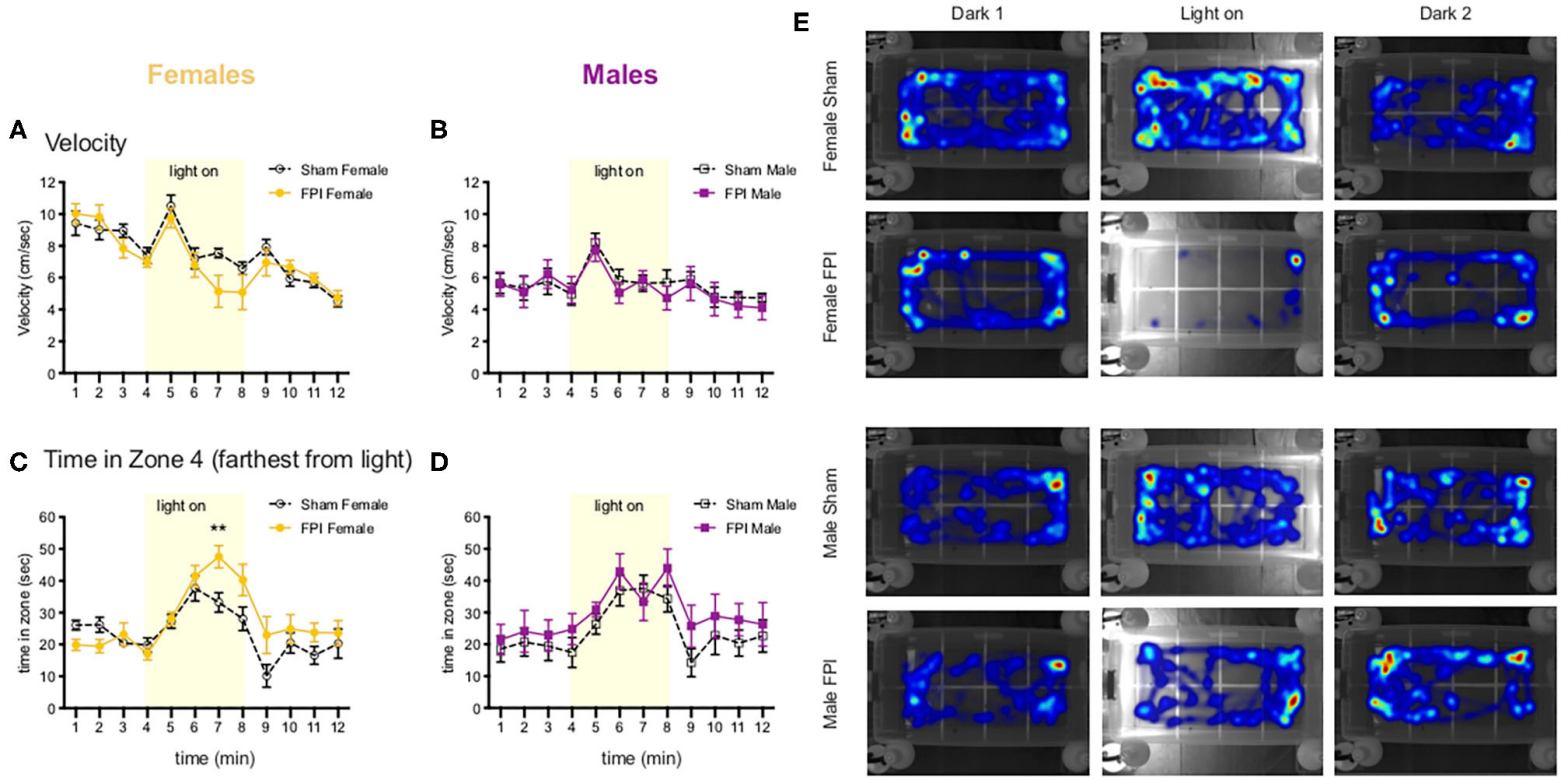
Females show photophobia-like response in the light gradient open field after FPI **(A,B)**. Change in velocity across the 12 min light gradient open field task. Females **(A)** move faster overall relative to males **(B)** throughout the task (*p* < 0.001 Female vs. Male for velocity). Note the peak in velocity for all groups after the onset of the light stimulus (min 5) **(C,D)**. Time spent in zone 4 (farthest from light), FPI females spent significantly more time in zone 4 during the light on phase (**p = 0.007 Sham Female vs. FPI Female). There were no significant differences among male groups. Data are represented as mean ± SEM; *n* = 9–10/group. **(E)** Representative heatmaps for each group across the 3 phases of the task. Note light on side was counterbalanced evenly across trials for each group.

In a subset of animals from the weak shocks experiment, we added a task to look at anxiety-like behavior and photophobia (light sensitivity) in a modified open field task with a sudden onset of a light gradient. Using this novel approach for application to our TBI model for photophobia-like behavior, after the first cohort we tested, we performed a *post hoc* power analysis with the program G^*^Power and found that at least n=8/group would provide sufficient statistical power at the recommended 0.80 level [surgery cohorts 2–3, group sizes were Sham Female, *n* = 9; FPI Female, *n* = 10; Sham Male, *n* = 10; FPI Male, *n* = 9 (due to one FPI male that jumped out of the open field and terminated the trial)]. The day after testing for contextual fear, we measured average velocity and zone preference across the 12 min task. A mixed factors ANOVA for sex, FPI, and time revealed a significant sex x time interaction [F(11,374) = 5.173, *p* < 0.001], where regardless of injury, females showed increased velocity during the dark phases compared to males (min 1–6 and min 9–11; female vs. male overall, *p* < 0.05; see Figures 6A,B). When we looked at zone preference, for time spent in zone 4 (farthest from the light) during the light-on phase (min 5–8) with a mixed factors ANOVA, we found a three way interaction for sex x injury x time [F(3,102) = 3.523, *p* = 0.018], where Female FPI rats spent significantly more time in zone 4 than Sham Female [min 7; t(17) = 3.049, *p* = 0.007, Sham Female vs. FPI Female; see Figure 6C]. There was no effect of injury between the male groups (Figure 6D). See representative heatmaps for each group across each phase of the task in Figure 6E.

#### Additional Analyses

**Figure 7:**
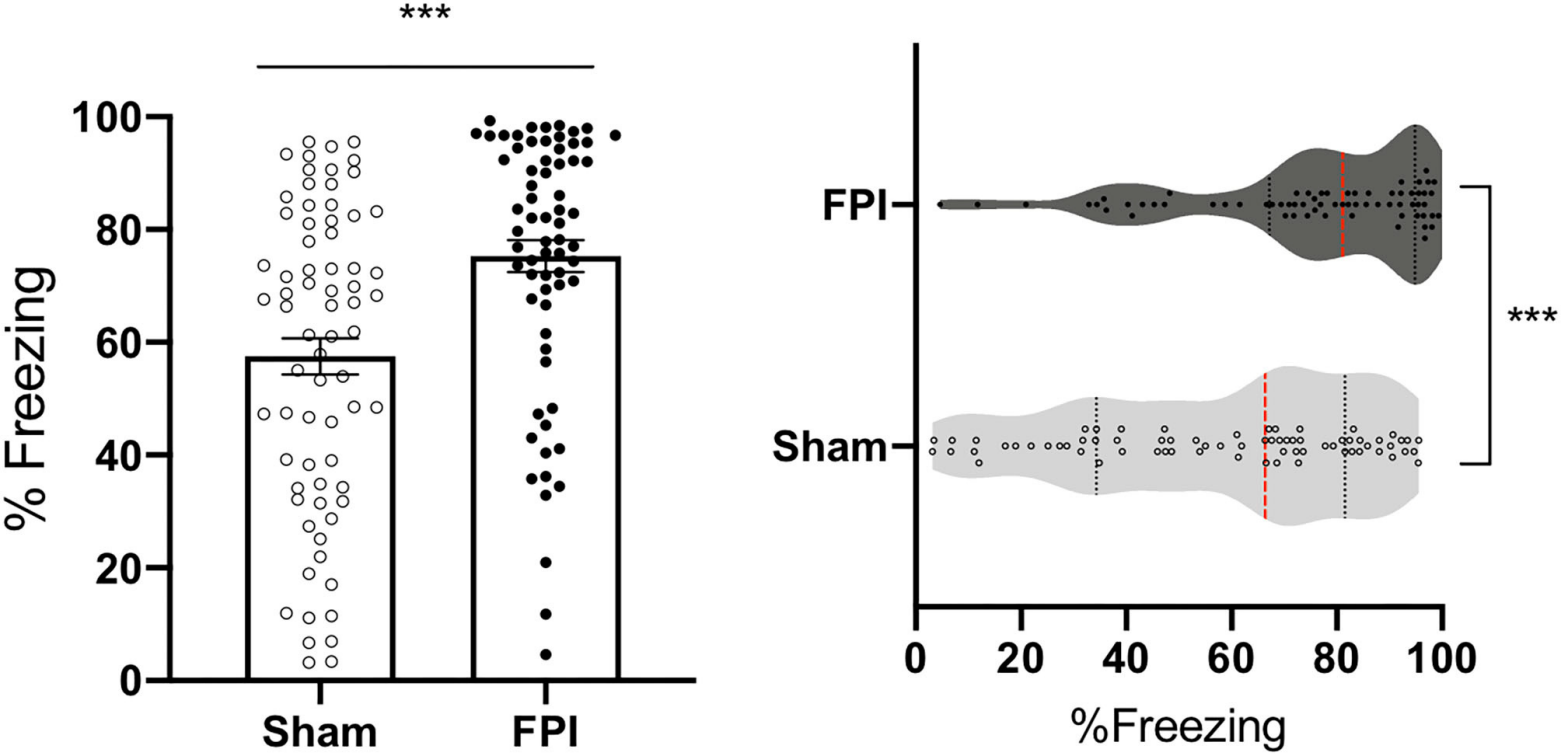
FPI enhanced context fear across all experiments. Across the three experiments, FPI groups showed significantly greater freezing in the conditioning context compared to sham (first 8 min), regardless of sex and shock intensity. Data from experiment 1 include recent context test only for consistency with the other experiments. Group totals: *n* = 71/Sham; *n* = 69/FPI. (Left) Data are represented as mean ± SEM; ***p < 0.001. (Right) Violin plots representing the distribution of the data for context fear, red dotted line represents the median and black dotted lines are quartile ranges;***p < 0.001.

The pattern in the data for the initial test of context fear memory, across experiments indicates that FPI leads to increased freezing to the context under 3 different conditioning protocols. Given this consistent pattern, we were interested in the overall effect of FPI on context fear irrespective of sex and varying fear conditioning parameters. Therefore, we performed an overall univariate ANOVA for sex and injury across all experiments for context fear (mean of 8 min). Data from experiment 1 include recent context test only for consistency with the other experiments represented. We found that there was a significant effect of sex [F(1,128) = 8.259, *p* = 0.005], where females tended to show reduced freezing to context, regardless of injury. We also saw a significant effect of experiment [F(2,128)=6.59, *p* = 0.002]. *Post hoc* analyses using Fisher's LSD test revealed that independent of sex and injury, rats in experiment 1 had the least amount of conditioning to the context relative to experiment 2A (white noise-shock vs. strong shocks alone; *p* = 0.007) and experiment 3 (white noise shock vs. weak shocks alone; *p* = 0.001). Finally, we found a significant main effect of injury on context fear [F(1,128) = 17.87, *p* < 0.001], suggesting that across all conditioning protocols FPI groups had a robust enhancement of contextual fear when tested the day after either auditory or unsignaled fear conditioning (Figure 7). An interesting observation in the data in Figure 7 visually reveal a wide and varying distribution of amount of freezing across all sham cohorts, however there is a skewed effect after FPI. Freezing levels tend to shift and cluster toward ceiling across FPI cohorts indicating that regardless of sex and fear conditioning parameters, FPI groups show elevated contextual fear.

## Discussion

The current study investigated the effects of TBI and sex on stimulus sensitivity and heightened defensive behaviors, which have clinical implications for comorbid anxiety, PTSD, and neurological complications. We found that diffuse TBI using the lateral fluid percussion injury model (FPI) led to rapid acquisition and enhanced context fear in both female and male adult rats. We also replicated our prior finding (30) that FPI caused auditory hypersensitivity to white noise, a phonophobia-like defensive behavior (freezing) in response to 75 dB white noise in males. The current study found that this phonophobia-like response was even more robust in FPI females. Additionally, we saw hypersensitivity to light, a photophobia-like behavioral response, in the light gradient open field task, where FPI females spent the least amount of time in the bright zone of the open field following a change in light stimulus in the task. The common sex differences in our data and the literature for these behavioral outcome measures are carefully considered in our interpretations in how TBI affects stimulus sensitivity, fear learning and memory, and defensive behaviors differently for females and males. While the severity of injury was balanced across females and males, sex by injury interactions are discussed to highlight the unique impact of TBI on females across physical and emotion related outcomes.

### Sex Differences

When interpreting interactions of sex and TBI, it is important to consider baseline differences and/or differences in control groups between the sexes on each endpoint measure. How females and males differ (and are similar) on behavioral defensive measures such as in fear conditioning and anxiety-like tasks will put into context the impact of TBI for each sex. In the current study we observed overall sex differences in context fear and velocity in the light gradient open field task. With strong footshocks (0.9 mA), males showed increased context fear in both auditory and context conditioning protocols. However, with weaker footshocks (0.5 mA), context fear was comparable between the sexes. This observation underscores the importance of stimulus parameters used in behavioral protocols, and where ceiling effects lie for each sex under each condition. Our work corroborates others in showing that adult females fear condition less to context (39–42), and locomote more in the open field (49, 50). Mechanistically, sex differences in fear induced analgesia have been reported where females exhibit less conditioned analgesia than males (51). These differences may play a role in shock sensitivity and influence fear memory encoding to nociceptive stimuli like shock, and importantly altered pain processing and interactions with TBI. Studies using human subjects have also found sex differences in response to heat and electric stimulation, with males showing higher pain tolerance than females (52). Baseline sex differences in physical, cognitive, and emotional domains are also prevalent in humans and carefully considered in the context of sports concussion (9, 13, 15), where females report more symptoms at baseline, even in the absence of a concussion. While males are at greater risk for TBI (4), mental health conditions such as anxiety and stress and trauma related disorders, as well as migraine and pain disorders are more common in women (28, 53). These potential vulnerabilities in females may represent risk factors for complications and comorbidities following TBI.

### General Effects of TBI: Heightened Defensive Behavior and Implications for Comorbid PTSD

An overall goal of our research remains to understand how stressful events and aversive stimuli are perceived and encoded following diffuse TBI. In our previous work (30) and the current study we continuously replicated that all TBI groups show a behavioral defensive response to 75 dB white noise that was not present in uninjured shams. In the previous study, we showed that FPI increased activity in auditory thalamo-amygdala projection neurons during white noise exposure. TBI-induced disruption to sensory processing pathways, and especially within sensory-emotional neurocircuits may be altered after injury and interpret otherwise neutral stimuli as aversive. For example, white noise comprises of the full range of sound frequencies and at high intensities is categorized as an audiogenic stressor (54–57). Future studies aim to determine what properties of noise drive this hypersensitivity after FPI as well as the post injury time course of the phonophbia-like response to white noise. Our prior work has consistently demonstrated enhanced fear learning following the fluid percussion injury model in male rats (30, 31). In the current study, we measured the behavioral consequences of TBI on fear learning and memory in females. The current study replicated the increased fear phenotype in additional fear conditioning protocols using unsignaled shocks of two different intensities. Importantly, we also found enhanced context fear in females after TBI. A related outcome that emerged in the current study was rapid acquisition of fear following unsignaled footshocks (see Figures 5A,D). Under both strong and weak shock intensity, both female and male FPI groups showed immediate freezing in the context following the first shock trial (pre-shock interval 2). This rapid defense response in both sexes may reflect enhanced fear learning, increased sensitivity to pain, or likely an interdependence of both interpretations considering pain and emotional sequelae early after TBI.

In behavioral experiments, we often find a normal spread of variability in learning and fear expression, such as in our context fear data for the uninjured controls (see Figure 7). Some factors tend to predict enhanced fear include premorbid anxiety-like behavior, prior stress exposure (58–61), and prior TBI [current study and (30, 31, 62)]. The relative difference is not necessarily always statistically significant in every cohort (see Figure 4A). However, when we consider multiple experiments under slightly different conditioning parameters, the pattern of enhanced context fear after FPI is consistent. It is translationally relevant to determine such risk factors common to both sexes that lead to a shift toward increased defensive behaviors representative of anxiety and fear to help inform the conditions under which PTSD might develop following trauma. In humans, one study of deployment related TBI revealed that although men were more likely to have a PTSD diagnosis, this effect was washed out when total blast exposure was accounted for (63).

### Sex by Injury Interactions: FPI Females Show Robust Sensory Sensitivity Across Multiple Modalities

The current study replicated our previous finding in that FPI produced a phonophobia-like defensive response (hypersensitivity to noise) to 75 dB white noise (30), and further found this effect was even more robust in FPI females. Furthermore, in a novel task to test defensive photophobia (hypersensitivity to light) in the light gradient open field (47, 48), we found FPI females exhibited a photophobia-like defensive response. These novel data have important implications for clinical concussion and TBI. Sensory sensitivity is a primary physical symptom of concussion and TBI, and our data reflect that females may be more impacted in this symptom domain after injury. These initial findings fit with the clinical epidemiology that females more often experience migraine, of which sensory sensitivity to light and noise are principal complications. Sex differences in candidate substrates such as calcitonin gene related peptide implicated in migraine (64) and post traumatic headache (65) may be involved in the affective component in sensory hypersensitivity after TBI and is the basis of future studies. In humans, a recent study of service members with TBI found females had higher total symptom scores, where sensitivity to light was among the most affected symptoms (66). Furthermore, this study also found that women with deployment related TBI had a higher incidence of somatosensory disturbances as well as depression with comorbid PTSD, owing to an elevated complexity of conditions after TBI (63). Interestingly, in a study in a pain clinic population, women reported higher pain-related frustration and fear (67), suggesting increased defense and negative emotions related to pain perception. Our data suggest that after TBI females are more sensitive to sensory stimuli across multiple modalities and this influences defensive behaviors like anxiety and fear. Future studies will address the neurobiological mechanisms that underlie these translationally relevant sex by injury interactions.

### Conclusions

TBI involves multidimensional sequelae that interact to often negatively impact physical and mental health. The consequences of brain trauma may impact and manifest in females and males differently. This was the first study to investigate the effects of sex and TBI on stimulus sensitivity and defensive behaviors related to anxiety and fear, with broad translational implications for increased risk for comorbidities like migraine, anxiety, and PTSD. These findings have implications for migraine, particularly post-TBI migraine/headache, although we didn't directly measure headache/pain, the phono- and photosensitivity are hallmark symptoms. Our study revealed that females were more affected by physical symptoms of TBI such as phonophobia and photophobia, which led to increased defensive behaviors. General sex differences in each outcome dependent on testing parameters should always be carefully considered in both experimental and clinical settings to avoid ceiling or floor effects that may occlude meaningful differences. While we are behind in our understanding of how sex uniquely impacts various consequences of TBI, fortunately there is growing awareness and momentum for the need to investigate sex effects of TBI pathophysiology and mental health. Our study adds to this growing literature supporting the practice of sex as a biological variable in animal models of disease such as in TBI and mental health.

## Data Availability Statement

The raw data supporting the conclusions of this article will be made available by the authors, without undue reservation.

## Ethics Statement

The animal study was reviewed and approved by the Animal Research Committee at UCLA.

## Author Contributions

AH designed and ran the experiments, analyzed the data, and wrote the manuscript. SW, AM, and AP helped run the experiments and assisted with data acquisition and manuscript preparation. SG helped run the experiments, and assisted with data acquisition and analysis and manuscript preparation. LF consulted on data analysis and assisted with manuscript preparation. CG helped design experiments, assisted with data analysis, and manuscript preparation. MF designed experiments, and helped with data analysis, organization, and manuscript preparation. All authors contributed to the article and approved the submitted version.

## Conflict of Interest

AH and CG have received past research funding from Avanir Pharmaceuticals. MF is director of research for Neurovation Labs. CG receives consulting fees from NFL-Neurological Care Program, NHLPA, serves on the advisory panel for the Major League Soccer, NBA, NCAA, US Soccer Federation, California State Athletic Commission, Highmark International, and has received speaker fees from Medical Education Speakers Network and book royalties from Blackwell Publishing: Prioritized Neurological Differential Diagnosis. The remaining authors declare that the research was conducted in the absence of any commercial or financial relationships that could be construed as a potential conflict of interest.
